# Tandem Tau and α-Synuclein Seed Amplification Assays Reveal α-Synuclein Copathology Potentiates Tau Seeding in Alzheimer’s Disease

**DOI:** 10.21203/rs.3.rs-10845149/v1

**Published:** 2026-09-04

**Authors:** Damian Gorski, Haley Evans, Michelle Pinho, Zaina Rehan, Matthew Perkins, Emile Pinarbasi, Jon Reader, Sami Barmada, Henry Paulson, Sandra Pritzkow, Claudio Soto

**Affiliations:** 1 Mitchell Center for Alzheimer’s Disease and Related Brain Disorders, Department of Neurology, University of Texas McGovern Medical School at Houston, Houston, TX, USA.; 2 The University of Texas MD Anderson Cancer Center UTHealth Graduate School of Biomedical Sciences, Houston, TX, USA.; 3 Michigan Alzheimer’s Disease Research Center, University of Michigan, Ann Arbor, MI, USA.

## Abstract

Accumulating evidence highlights the frequent co-occurrence of tau and α-synuclein (αSyn) pathology across neurodegenerative diseases, including Alzheimer’s disease (AD) and synucleinopathies. Whether the co-deposition of these proteinopathic aggregates influences their respective seeding capacities remains unknown. Seed amplification assays (SAAs) enable sensitive and specific detection of pathological seeding activity in brain tissue and cerebrospinal fluid (CSF) and have been widely used to assess tau or αSyn seeding independently; however, their tandem application in the same individuals has not been thoroughly investigated. Here we apply Tau- and αSyn-SAA in parallel on a well-characterized postmortem cohort of matched brain tissue and CSF spanning AD, synucleinopathies, other neurological diseases, and neuropathologically defined controls. We demonstrate that tau seeding activity in both brain and CSF closely reflects underlying neuropathological burden, increasing stepwise with the grade of AD neuropathological change (ADNC) and neurofibrillary tangle (NFT) stage, with strong within-donor concordance between matrices (*r* = 0.79, *p* < 0.001). Tau-SAA measured in postmortem CSF robustly discriminated AD-related neuropathology from other groups, supporting its potential as a neuropathologically informed biomarker. αSyn-SAA identified all synucleinopathy donors as positive and revealed that 44% of AD donors harbor concomitant αSyn seeding activity. Leveraging this tandem dataset, we find that αSyn-SAA positive AD donors exhibit significantly enhanced tau seeding activity in both brain and CSF, independent of NFT stage, age, and sex (*p* = 0.014). In synucleinopathy donors, tau seeding did not alter αSyn seeding capacity (*p* = 0.606). Together, these results suggest a potentially unidirectional relationship between tau and αSyn seeding in human disease, wherein αSyn copathology acts as a catalyst of tau seeding in AD. Our findings carry implications for understanding how mixed proteinopathies shape disease burden and trajectory and may represent the beginning of a new direction in the field moving towards precision medicine to identify all types of pathology concomitantly affecting the brain, which will have a profound impact on patient prognosis and treatment.

## Introduction

Many aging-related neurodegenerative diseases are increasingly recognized as proteinopathies, in which specific proteins misfold, aggregate, and accumulate as amyloid deposits in the brain (1, 2). Tau and α-synuclein (αSyn) are the defining proteins implicated in two of the most prevalent proteinopathy classes: tauopathies, including Alzheimer’s disease (AD), and synucleinopathies, which encompass Lewy body disease (LBD) and multiple system atrophy (MSA) (2, 3). In AD, hyperphosphorylated tau accumulates into neurofibrillary tangles (NFTs) that closely track regional neurodegeneration and cognitive decline (4–7). In synucleinopathies, misfolded αSyn accumulates as neuronal Lewy bodies in LBD or glial cytoplasmic inclusions in MSA (8–10).

Although it is tempting to conceptualize tau and αSyn pathologies as mutually exclusive and not interacting, recent evidence suggests pure pathologies may be the exception rather than the rule (2, 11). Autopsy studies reveal that 40–60% of pathologically confirmed AD cases concurrently harbor αSyn pathology (12, 13). Likewise, tau pathology appears similarly prevalent in LBD, in which approximately 50% of patients diagnosed with Parkinson’s disease (PD), dementia with Lewy bodies (DLB) and Parkinson’s disease dementia (PDD) display tau pathology at a level equal to or greater than Braak stage III (14, 15). In MSA, the prevalence of multimorbidities remains somewhat unclear (16, 17), although recent studies have highlighted the presence of AD copathology including hyperphosphorylated tau in circumscribed brain regions (18).

The co-occurrence of multiple proteinopathies may in part explain the vast clinical heterogeneity observed in neurodegenerative disease, and undoubtedly complicates diagnosis, treatment, as well as the design and testing of new therapies (19). Moreover, recent lines of evidence suggest that tau and αSyn copathology may be associated with heightened disease severity and less favorable outcomes (20, 21). In longitudinal studies, AD patients with αSyn copathology exhibited accelerated disease progression and cognitive decline (22–24). Using longitudinal tau PET measures and linear regression analysis, Franzmeier et al, proposed that concomitant αSyn may potentiate amyloid-driven tau accumulation in AD (25). Congruently, cognitive assessment scores deteriorated faster in clinically diagnosed DLB patients with AD copathology relative to those without (26), suggesting that tau contributes to dementia in at least certain synucleinopathies.

These measurable clinical associations raise the possibility of synergistic interactions between co-occurring proteinopathies including tau and αSyn (27). Several studies in animal models have suggested that αSyn copathology influences the progression and spreading of tau (28, 29). Bassil and colleagues demonstrated that simultaneous introduction of AD brain-derived tau along with αSyn preformed fibrils markedly increased tau spreading relative to AD brain alone and found that this effect was attenuated in the absence of endogenous αSyn (28). Notably, the presence of tau seemed to have no effect on αSyn accumulation, suggesting a unidirectional relationship in which αSyn potentiates tau propagation but not the converse. Whether this asymmetry is recapitulated in human disease remains unclear.

The propagation of both tau and αSyn is thought to occur through a prion-like seeding-nucleation mechanism in which the misfolded protein conformer templates its disease-associated structure onto the natively folded form, driving pathological spread throughout the brain (1, 3). This self-replicative property can be directly exploited using seed amplification assays (SAAs), in which patient-derived biological samples are used to nucleate the aggregation of recombinant monomeric αSyn, provided as the reaction substrate (30). αSyn-SAA has emerged as a highly sensitive and specific tool for the detection and diagnosis of synucleinopathies using antemortem cerebrospinal fluid (CSF) (30–34). An analogous assay for the detection of tau seeds (Tau-SAA) has shown promise in brain tissues but remains comparatively less characterized and the detection of tau seeds in CSF remains elusive (35–38). While Tau- and αSyn-SAAs have been used independently, their tandem application, which enables within-donor comparison of tau and αSyn seeding, has not yet been thoroughly explored. Moreover, whether the presence of comorbid pathology influences the seeding capacity of the primary proteinopathy remains unknown.

Here we apply Tau- and αSyn-SAA in tandem on a well-characterized postmortem cohort of matched brain tissue and CSF to interrogate the relationship between tau and αSyn seeding in human disease. We first systematically characterize the performance of both assays, establishing their fidelity against underlying neuropathological measures. We then leverage this paired dataset to examine whether concomitant tau or αSyn pathology influences the seeding capacity of the primary proteinopathy, and whether this relationship remains consistent across disease contexts. Our results provide evidence that αSyn copathology may be an active modifier of tau seeding in AD in humans. Interestingly, the reciprocal relationship does not appear to extend to synucleinopathies. As tau seeding is intricately linked to its accumulation and capacity to propagate throughout the brain, these findings carry implications for understanding how αSyn copathology may shape disease trajectory in AD.

## Results

### Overview of study cohort

To investigate both tau and αSyn seeding activity within donors we performed parallel Tau- and αSyn-SAA on paired postmortem brain tissue and CSF (Fig. 1A). We first assembled a cohort of 82 frontal cortex human brain samples with paired CSF for 79 cases (**Supplementary Fig. 1A**). Across the entire cohort, mean age at death was 71.2 ± 16.8 years and 59.8% of donors were male (49/82). Clinical diagnoses spanned 14 distinct categories, including probable AD, Lewy body disorders, other neurodegenerative diseases, as well as cognitively normal healthy controls. Upon postmortem brain examination, cases were assigned to one of 11 neuropathological diagnoses (**Supplementary Fig. 1B-C**). All neuropathological evaluations included assessment of AD neuropathological change (ADNC) in accordance with NIA-AA ABC scoring criteria (39), which integrates amyloid-β deposition (Thal phase, A), staging of neurofibrillary tangles (NFT stage, B), and scoring for neuritic plaques (CERAD, C) to yield a composite ADNC designation. All samples were additionally screened (anterior cingulate and midbrain) and staged for Lewy body pathology and examined for other co-morbid pathologies (**Supplementary Fig. 1A, D**).

To facilitate analysis, cases were subsequently binned into four groups, AD (*n* = 36), synucleinopathies (SYN, *n* = 23), other neurological disease (OND, *n* = 12), and healthy controls (HC, *n* = 11) based on their primary neuropathological diagnosis (Fig. 1B, **Supplementary Fig. 1E**). Expectedly, donors differed in burden of AD-related and Lewy body pathology across groups (**Supplementary Fig. 2C-G)**. Groups also differed in age and sex distribution. AD and SYN donors presented with more advanced age at death compared to HC (*adj p* < 0.01). The AD group had a higher proportion of female donors relative to SYN and HC groups (*adj p* < 0.05). Notably, all HC donors were male (**Supplementary Fig. 2A-B**). Complete cohort and group level demographic and neuropathological characteristics are provided in **Supplementary Table 1.**

### Tau seeding activity increases with neuropathological severity

We next assessed tau seeding capacity across brain tissue samples from all donors. Brain homogenates (10% w/v; BHs) were diluted in a ten-fold series (from 10^−4^ to 10^−10^) and used to seed Tau-SAA reactions. Individual replicates were considered positive if fluorescence exceeded an empirically defined threshold of 1000 au (~20 × background). To compare the extent of seeding across samples, we derived a Tau seeding score (Tau-SS) as the sum of the proportion of positive replicates across all dilutions (Fig. 1C). The extent of tau seeding varied significantly across groups (Kruskal-Wallis, *p* < 0.0001). Tau seeding in the AD group significantly exceeded all other groups (*adj p* < 0.01, Dunn’s post-hoc with Benjamini-Hochberg correction). Interestingly, SYN donors showed elevated tau seeding relative to HC (*adj p* = 0.007). Overall, both OND and HC donors showed similarly low tau seeding activity consistent with minimal or absent tau pathology in these groups (Fig. 1D, **Supplementary Fig. 3A**). Stratifying donors by ADNC revealed a stepwise increase in tau seeding with more advanced ADNC (Jonckheere-Terpstra test, *JT* = 1861, *p* < 0.0001, Fig. 1E). This relationship was even stronger when stratifying by NFT-stage alone (JT = 2050, *adj p* < 0.0001, Fig. 1F), consistent with tau pathology being the primary driver of tau seeding.

To validate our Tau-SS as a semi-quantitative measure of seeding activity, we calculated the 50% seeding dose (SD50; dilution that yields 50% positive replicates) using the Spearman-Kärber method, which has been previously used to quantify seeding activity (40). The Spearman-Kärber method requires at least one dilution at which all replicates are positive, thus rendering most OND and HC donors as unquantifiable and limiting quantification to 42 of 82 samples (**Supplementary Figure 3B**). Nevertheless, SD_50_ correlated near-perfectly with Tau-SS among quantifiable samples (Pearson’s *r* = 0.999, *p* < 0.001, **Supplementary Fig. 3C**), confirming Tau-SS as a robust and more inclusive measure of tau seeding capacity.

### CSF Tau-SAA reflects brain tau seeding and discriminates AD neuropathology

Having established that the extent of tau seeding activity in brain tissue reflects neuropathological burden, we next examined whether Tau-SAA could detect seeding in CSF, given its potential relevance as a diagnostic biomarker matrix. CSF samples collected postmortem were seeded neat and in three-fold dilution steps (1:3 – 1:729). Analysis of neat samples alone revealed positive tau seeding activity (at least three of five replicates surpassing 1000 au threshold) in the majority of AD group donors (82%) but not in healthy control donors (HC: 0%). Positivity rates increased stepwise with respect to increasing ADNC and NFT burden (**Supplementary Fig. 4A**). To better capture differential tau seeding magnitudes between individual samples, we similarly derived a CSF Tau-Seeding Score by summing the proportion of positive wells across the full dilution series (Fig. 2A). Congruent with our brain tissue analysis, CSF seeding was most robust in AD donors (*adj p* < 0.01) and collectively SYN donors demonstrated elevated seeding relative to HC (*adj p* = 0.02, Fig. 2B). Jonckheere-Terpstra trend analysis demonstrated CSF tau seeding increased with progressively greater ADNC (JT = 1676.5, *p* < 0.0001) and NFT stage (JT = 1918.5, *p* = 0.0001, Fig. 2C–D). To determine whether tau seeding activity in CSF accurately reflects that observed in brain tissue, we performed within-donor correlation analysis between brain and CSF Tau-SS (*n* = 79 paired samples). Seeding across matrices appeared strongly correlated (*r* = 0.79, *p* < 0.0001), demonstrating high concordance within subjects (Fig. 2E).

The ability of CSF Tau-SAA to faithfully reflect brain pathology supports its utility as a potential AD biomarker. To further explore this notion, we performed receiver operating characteristic (ROC) analysis using CSF Tau-SS as a continuous predictor. CSF Tau-SAA robustly discriminated AD from controls (AUC = 0.95) with 89% sensitivity and 100% specificity (Fig. 2F, **Supplementary Fig. 4B**). Discrimination remained strong (AUC = 0.86) when including other neurologically affected groups, reflecting a more clinically relevant scenario in which multiple diagnoses might be considered. Likewise, CSF Tau-SAA readily separated donors according to neuropathological measures, distinguishing those with high or intermediate ADNC (AUC = 0.92), and advanced NFT-stage (B2/B3; AUC = 0.92) from those without. Notably, at optimal thresholds, CSF Tau-SS identified donors with advanced ADNC with 90% sensitivity and 88% specificity. Discrimination of advanced NFT burden was particularly robust, with 97% specificity, 80% sensitivity, and 97% positive predictive value, suggesting that Tau-SAA seeding in postmortem CSF is a reliable indicator of significant NFT pathology (**Supplementary Fig. 4B**).

### αSyn-SAA identifies synucleinopathies and reveals frequent αSyn copathology in AD

To complete our integrated SAA panel, we performed αSyn-SAA on all 82 brain samples and 79 paired CSF samples (**Supplementary Fig. 5A-B**). Brain homogenates were seeded across an abbreviated ten-fold dilution series (from 10^−6^ to 10^−10^, Fig. 3A) and CSF samples processed neat given the established sensitivity of αSyn-SAA. One CSF sample yielded inconclusive results (only 66% of wells were positive, even after retesting) and was removed from downstream analysis. Of synucleinopathy cases (*n* = 23), 100% tested positive by CSF αSyn-SAA (Fig. 3B). Positivity rates were markedly lower among OND (9%, 1/11) and HC donors (10%, 1/10). Among AD donors, 44% (15/34) of cases tested positive by αSyn-SAA, consistent with figures reported in other studies (13). Interestingly, neuropathological analysis of these patients showed only 6/36 (17%) positive for Lewy bodies (**Supplementary Table 1**). When stratified by neuropathological diagnosis, 100% of donors with LBD (*n* = 15) and MSA (*n* = 8) tested αSyn-SAA positive in CSF (Fig 3C). With the exception of AD (44%, 15/34), positivity rates in other diagnoses were otherwise low (HD: 25%, 1/4; HC 10%, 1/10).

Brain αSyn-SAA was remarkably consistent with results observed in postmortem CSF. Brain αSyn-SAA seeding scores (Brain αSyn-SS) were significantly elevated in SYN group donors relative to all other groups (*adj p* < 0.0001, Fig. 3D). Moreover, donors determined as CSF αSyn-SAA positive demonstrated significantly higher levels of brain seeding relative to their negative counterparts (*p* < 0.0001, Fig. 3E), confirming a high degree of concordance between CSF and brain αSyn seeding measures. Together, these results suggest αSyn-SAA reliably captures αSyn seeding activity across both brain and CSF matrices and demonstrate that a substantial proportion of AD donors harbor concomitant αSyn pathology detectable by SAA.

### Comorbid αSyn enhances tau seeding in AD

With robust tau and αSyn seeding measures established across both brain and CSF, our tandem SAA panel uniquely enabled within-donor examination of cross-pathology interactions. Previous studies have suggested concomitant αSyn may modulate tau spreading (28) and influence tau accumulation in AD (25, 27, 41). To explore whether this paradigm likewise extends to seeding capacity, we first isolated AD group donors (*n* = 34) and stratified them by CSF αSyn-SAA status (SAA+ = 15, SAA− = 19). Groups were largely comparable in terms of age at death (Wilcoxon *p* = 0.716), sex distribution (χ^2^ *p* = 0.82), and NFT stage (Fisher’s *p* = 0.37).

To specifically examine the effect of αSyn-positivity on tau seeding in AD, we applied a linear mixed model controlling for the matrix type (brain or CSF), NFT stage, age at death, sex, and a donor-specific random effect. Compared with their negative counterparts, αSyn-SAA+ AD donors exhibited significantly greater tau seeding (β = 1.076, *p* = 0.014). Expectedly, NFT stage was the strongest predictor of tau seeding (β = 2.18, p = 0.001), while age and sex failed to reach significance after adjustment (Fig. 4A–C, **Supplementary Table 2**). The effect of αSyn positivity on tau was consistent irrespective of which matrix was used to assess tau seeding with no significant interaction between matrix type and αSyn-SAA status (β = 0.295, *p* = 0.61, **Supplementary Table 3**). Independent linear models for brain and CSF confirmed αSyn-positivity as a significant predictor in both matrices (brain: β = 0.837, p = 0.020; CSF: β = 1.315, p = 0.040; **Supplementary Table 4**). Collectively, these findings suggest that comorbid seeding-competent αSyn pathology is associated with enhanced tau seeding propensity in AD patients independent of NFT stage, age, and sex.

### Tau does not influence αSyn seeding in synucleinopathies

To investigate the reciprocal relationship, we next examined whether secondary tau pathology similarly enhances αSyn seeding within SYN group donors (*n* = 23). Consistent with known differences in pathological αSyn burden and neuroanatomical distribution between LBD and MSA (42, 43), brain αSyn seeding was significantly higher in LBD compared to MSA donors (Wilcoxon *p* = 0.005, Fig. 5A). Notably, concomitant AD pathology was more prevalent in LBD versus MSA donors, with 53% (8/15) of LBD donors showing intermediate or high ADNC, typically considered sufficient to contribute to cognitive impairment (39), compared to none (0/8) in MSA. Likewise, tau seeding activity trended higher in LBD relative to MSA but did not reach significance (Fig. 5B–C). Although unadjusted correlation analysis suggested a modest positive trend between αSyn and tau seeding (*r* = 0.229, *p* = 0.293, Fig. 5C), this association was attenuated after accounting for neuropathological diagnosis (β = 0.092, *p* = 0.606), suggesting that this trend reflects diagnosis-level differences rather than a direct effect of tau on αSyn seeding capacity.

Together, these results are consistent with an asymmetric relationship between tau and αSyn pathology, whereby αSyn copathology enhances tau seeding in AD but tau copathology seemingly does not influence αSyn seeding in synucleinopathies.

## Discussion

Accumulating evidence suggests copathology may modulate disease trajectory. Here we set out to determine whether comorbid pathology influences one of the central tenets of neurodegenerative disease: the ability to seed or self-propagate the pathological structure onto natively folded protein. We utilized a novel paired cross-matrix approach, applying both Tau- and αSyn-SAA, along with a semi-quantitative serial-dilution titration, to measure tau and αSyn seeding activity in matched brain tissue and CSF. Our central finding is that αSyn-SAA positive AD donors exhibit significantly enhanced tau seeding activity, suggesting that αSyn is not merely a passive bystander, but instead a meaningful catalyst of tau propagation in AD.

As a foundation for our downstream analysis, we first examined the relationship between tau seeding and underlying tau pathology in the brain. Tau seeding in brain tissue readily discriminated AD donors from all other groups. Seeding was also elevated in synucleinopathy donors relative to controls, consistent with the slightly higher NFT stage observed across these samples. Our analysis of 82 brain samples spanning all ADNC classifications and NFT stages revealed a strong correlation between tau seeding, AD pathology and more specifically tau NFT stage. These findings complement and expand upon a similar study by Frey et al, who noted that brain homogenates from higher Braak stage donors display faster aggregation kinetics and correlate with brain phosphorylated tau levels (36). Our serial dilution approach extends this observation further and demonstrates that donors with more advanced tau pathology retain seeding activity at higher dilutions, suggesting a greater abundance of seeding-competent species.

While the detection of proteinopathic seeding in brain tissue represents an important first step, the intra vitam detection of such seeds in readily accessible biofluids holds far greater clinical utility for antemortem diagnosis. Although CSF SAAs are readily utilized for the diagnosis of synucleinopathies and prion diseases (30, 31, 44), the detection of tau seeding in antemortem CSF remains a critical gap in the field. Sajio and colleagues provided initial evidence that a 4R-Tau SAA can detect seeding in antemortem CSF from 4R-tauopathies but noted highly diminished seeding relative to CSF collected postmortem and brain tissues (38). A later study utilizing tau biosensor cells found no detectable tau seeding in antemortem AD CSF (45), whereas another employing a nanoluciferase enzyme complementation strategy noted antemortem CSF tau seeding but struggled to distinguish AD from control CSF (46). Our postmortem CSF results provide an important intermediate step towards antemortem detection of tau seeding. We find that CSF Tau-SAA demonstrates robust and reproducible seeding that faithfully tracks underlying ADNC and tau neuropathological burden and strongly correlates with brain tissue tau seeding activity. These results provide a strong rationale for the future development and assessment of Tau-SAA in antemortem biofluids, where it may serve to complement existing phosphorylated tau biomarkers by capturing seeding-competent tau as a functionally distinct dimension of AD-related pathological burden.

Consistent with prior reports, αSyn-SAA demonstrated high sensitivity for synucleinopathies (26, 32, 34, 47). CSF analysis identified all LBD and MSA samples as positive and accordingly displayed stronger αSyn seeding in brain tissue. Among AD donors, 44% tested αSyn-SAA positive in CSF, consistent with previously published rates (12, 13), yet notably exceeding the ~17% of AD donors with LBD pathology identified by neuropathological examination. This discrepancy highlights that αSyn-SAA detects and amplifies seeds beyond the threshold of histological evaluation and is consistent with other observations that SAA exceeds the sensitivity of immunohistochemistry (48).

Considering this substantial subset of αSyn-SAA+ AD donors, we next explored whether the presence of αSyn copathology influences tau seeding. Previous clinical investigations have determined αSyn-SAA positivity may impart detrimental effects on cognition in AD patients and accelerate symptomatic progression on standardized cognitive assessments over time (22–24). Ding and colleagues reported that αSyn-SAA positivity was associated with increased risk of mild cognitive impairment (MCI) to AD conversion and suggested a possible role of αSyn in tau-related pathology based on strongly correlated αSyn and tau levels (49). Franzmeier and colleagues explored this association further, demonstrating that αSyn-SAA positive AD patients exhibited elevated CSF pTau-181, greater tau-PET tracer signal, and faster Aβ-driven tau accumulation, with αSyn-SAA positivity additionally predicting cognitive decline (25). While tau-PET and pTau effectively measure tau accumulation and phosphorylation, respectively, here we take a complementary approach by directly measuring tau seeding in postmortem brain and CSF. Expanding on these previous observations, we find that αSyn-SAA positive AD donors exhibit significantly enhanced tau seeding activity after controlling for NFT stage, age, and sex. Our data suggest that the potentiating effect of αSyn on tau pathophysiology extends to its seeding capacity, a property intimately linked to, albeit distinct from, tau propagation and accumulation.

We hypothesize that the enhanced tau seeding activity observed in αSyn-SAA positive AD donors may reflect either a greater abundance of seeding-competent tau species or an increase in the intrinsic seeding potency of tau seeds themselves in the presence of αSyn copathology. Notably, these possibilities are not mutually exclusive and could plausibly act in concert to influence tau seeding. Furthermore, even an effect driven primarily by greater abundance raises the question of how αSyn copathology promotes tau accumulation. One possibility is that αSyn codeposition changes the cellular environment, altering kinases/phosphatases levels, disrupting proteostasis and impairing clearance mechanisms (50, 51). Others have postulated a more direct relationship in which αSyn initiates tau fibrillization through a cross-seeding mechanism, thus modulating tau accumulation (52, 53), although direct evidence for such cross-seeding in human disease remains limited.

Alternatively, αSyn copathology may shape the inherent properties of tau seeds, such as their conformation, posttranslational modifications (PTMs) or assembly state in ways that enhance their seeding competency. Ample evidence suggests tau seeding efficiency is governed by aggregate structural conformations (54, 55). While the tau filament structure from the AD brain has recently been solved (56), it remains unknown whether the AD tau-fold is modified by αSyn codeposition. Kasen and colleagues demonstrated that PTMs and particularly phosphorylation at key residues modulate tau seeding competence (57). Importantly, others have demonstrated distinct PTM molecular signatures in AD with and without LBD pathology, suggesting that αSyn comorbidity can influence tau PTMs (58) and thus plausibly modify intrinsic seeding capacity. Collectively, whether αSyn modulates tau seeding competency through shifts in conformation, PTM profile, molecular assembly state or some combination thereof remains an important open question that future biochemical and structural studies are well-positioned to address.

To investigate whether the relationship between tau and αSyn seeding is bidirectional, we conducted an exploratory analysis of whether tau seeding alters αSyn seeding in synucleinopathy donor brain tissue. Our analysis suggests tau has no meaningful effect on αSyn seeding capacity, consistent with studies in animal models by Bassil and colleagues in which αSyn unidirectionally potentiated tau pathology in the absence of a reciprocal effect (28). Despite this apparent congruency, we interpret this null result with caution considering the modest sample size (*n* = 23) and the use of frontal cortex brain tissue in which αSyn pathology is relatively sparse across both LBD and MSA (59). These constraints may have limited our ability to detect subtle tau-driven effects on αSyn seeding. Future studies examining larger cohorts and additional brain regions, such as the substantia nigra and limbic cortex, are needed to more thoroughly assess the directionality and disease specificity of tau-αSyn seeding interactions.

We acknowledge several limitations of our study. Our principal finding that αSyn-SAA positive AD donors exhibit enhanced tau seeding is associative in nature and does not directly imply a causal relationship. Additionally, the studies performed herein utilized brain tissue and CSF from deceased donors and as such represent a single time point and a confined brain region. Postmortem CSF may imperfectly reflect certain characteristics of that collected antemortem, such as protein concentration, degradation, and may be unduly affected by the postmortem interval. Finally, certain analyses were limited by sample size and would benefit from future validation with larger cohorts.

Taken together, our findings underscore the complexity of mixed proteinopathies in neurodegenerative disease and highlight the value of characterizing the seeding activity of multiple analytes within the same individual. Looking forward, the continued development of sensitive and validated antemortem biofluid SAAs for tau and other disease-defining proteins such as TDP-43 could enable comprehensive proteinopathy profiling. This type of multi-analyte SAA panel could not only improve diagnostic precision and patient stratification but also inform clinical trial design and the development of targeted therapeutic strategies tailored to an individual’s specific proteinopathic burden.

## Methods

### Human samples and compliance with ethical standards

Frontal cortex (BA9–10) brain tissue and cerebrospinal fluid (*n* = 72 paired samples) were obtained from the University of Michigan, Michigan Alzheimer’s Disease Research Center and Michigan Brain Bank, part of Michigan Medicine. Additional samples (*n* = 10) were obtained from the Boston University Alzheimer’s Disease Research Center and Boston University CTE Center. All samples underwent detailed neuropathological evaluation by a board-certified neuropathologist. All samples utilized in this study were acquired from deceased and de-identified individuals with informed written consent for brain autopsy and use of materials for research purposes.

### Collection and Processing CSF

Prior to brain removal, postmortem CSF was carefully extracted from the lateral ventricles using a luer-lock syringe. CSF was transferred to 15 mL polypropylene conical tubes and placed on wet ice until processing. Postmortem CSF was centrifugated at 400g for 15min at 4°C, aliquoted and stored at −80°C until further use.

### Group assignment criteria

All human donors underwent detailed postmortem neuropathological evaluation by a board-certified neuropathologist. Each donor was assigned a primary neuropathological diagnosis by the reviewing neuropathologist, reflecting the pathology judged most likely to have driven clinical presentation. This primary neuropathological diagnosis was used to assign donors to one of four experimental groups: Alzheimer’s disease (AD), synucleinopathy (SYN), other neurological disease (OND), or healthy control (HC). Donors with a primary neuropathological diagnosis of AD including one case of Down syndrome with AD (DSAD; *n =1*) were assigned to the AD group. LBD and MSA donors were collectively assigned to the SYN group. Other neurodegenerative diagnoses excluding AD and Synucleinopathies (ALS, HD, MS, SCA3, etc) were assigned to the OND group. Donors without significant neuropathological findings were considered HC. Additional neuropathological findings and comorbidities were considered secondary to the primary neuropathological diagnosis and were not considered for group assignment.

All samples were evaluated for AD neuropathological change (ADNC) according to the 2012 National Institute on Aging – Alzheimer’s Association guidelines for the neuropathologic assessment of AD (39). ADNC “ABC” scores were determined on the basis of three constituent parameters: amyloid-β plaque score (Thal phase, A), neurofibrillary tangle stage (Braak stage, B) and CERAD neuritic plaque score (C). All cases were screened for Lewy pathology on sentinel sections (midbrain and anterior cingulate) and staged according to consensus criteria established by the DLB Consortium (60). Neuropathologic evaluation was performed on one hemisphere, primarily left, while the other was flash frozen for biochemical studies.

### Preparation of brain homogenate

Frozen brain samples were carefully minced with a scalpel and placed in 2 mL Precellys CK14 soft tissue homogenization tubes. Ten percent (w/v) brain homogenates were prepared in phosphate-buffered saline (PBS, Cytiva), supplemented with 1x EDTA-free protease inhibitor (Roche) and 1x phosphatase inhibitor, using a Precellys tissue homogenizer.

### Recombinant protein substrate expression and purification

Expression and purification of human Tau substrate were performed as previously described (35). Succinctly, A pET-28b expression vector encoding human full-length and cysteine-free 0N3R tau was transformed and expressed in BL21(DE3) *E. Coli* (New England Biolabs). Starter cultures were diluted into 500 mL of Terrific Broth auto-induction media (Formedium) and grown overnight. Bacteria were harvested by centrifugation, resuspended, and lysed with sonication (Misonix 3000). Lysates were boiled for 30 min at 95 °C, and centrifuged again. Soluble proteins were salted out with ammonium sulfate (55% w/v). Precipitated proteins were collected by centrifugation and stored at −20 °C until use. Tau protein was purified using a one-step cation exchange chromatography protocol. Eluted fractions were assessed by SDS-PAGE, and those containing tau were dialyzed twice against 100 volumes of 10 mM Hepes pH 7.4 and 100 mM NaCl. Dialyzed protein was filtered through a 100 kDa molecular weight cut off (MWCO; Millipore Sigma) and the permeate aliquoted, snap frozen, and stored at −80 °C.

Monomeric αSyn substrate was expressed and purified as previously reported (31, 33). In short, C-terminally His-tagged human αSyn was expressed in BL21(DE3) pLysS (Invitrogen) *E. Coli* cells using 0.1 mM IPTG (isopropyl β-d-thiogalactoside). Bacteria were collected and lysed in 50 mM NaH_2_PO_4_ (pH 8.0), 300 mM NaCl, 10 mM imidazole, 1 mM PMSF, 0.1 mM tris-(2carboxyethyl) phosphine (TCEP) and 1 mg ml^−1^ lysozyme followed by intermittent probe sonication. The resulting lysate was precleared (12,000*g* for 15 min at 4 °C) followed by ultracentrifugation at 100,000*g* for 30 min at 4 °C. The supernatant was loaded onto a nickel-affinity column (Sepharose fast flow, Cytiva) and bound protein eluted using 250 mM imidazole. αSyn containing fractions were selected for dialysis against PBS followed by 100 kDa cut-off filtration. Protein was pooled, aliquoted and stored until further use.

### Tau seed amplification assay (Tau-SAA)

Tau-SAA was performed as previously described (35) with some modifications. Briefly, tau substrate was thawed and adjusted to 20 μM in a reaction mix consisting of 10 mM Hepes pH 7.4, 10 μM Thioflavin T (ThT), 175 μM NaCl, 0.1 mg/mL bovine serum albumin (BSA), and 120 μM Heparin. To prepare Tau-SAA reactions, 63 μL of reaction mix was dispensed into each well of a black, clear-bottom, nonbinding polystyrene 384-well microplate (Corning). Brain homogenates or CSF samples were allowed to thaw on ice, diluted in 10 mM Hepes pH 7.5 with 100 mM NaCl or seeded neat into designated wells, yielding a final volume of 70 μL per well. Five to six replicates were utilized for each sample. Reactions were thoroughly mixed and then sealed with Masterclear real-time PCR film (Eppendorf). Prepared Tau-SAA plates were placed inside an Omega microplate reader (BMG Labtech) set to 47 °C and subjected to intermittent cycles of orbital shaking (800 rpm) for one min every hour. ThT fluorescence was recorded after each shaking cycle (λ_ex_/λ_em_ = 440/490 nm). Reactions were terminated after 120 hours

### Alpha-synuclein (αSyn) seed amplification assay

αSyn-SAA was performed as outlined in previous publications (32, 61). For brain tissue homogenates, samples were diluted and 40 μL were added to a clear-bottom 96-well microplate (Greiner) along with a reaction mix (60 μL) consisting of 0.2 mg/ml recombinant 6xHis αSyn monomeric protein, 100 mM PIPES pH 6.5 (Sigma-Aldrich), 500 mM NaCl, 10 μM ThT, along with two 1/8-inch silicon nitride beads (Xiamen MasCera Technology). Amplification reactions were performed in an Omega microplate reader at 42 °C with 1 min of orbital shaking (800 rpm) every 14 minutes. Fluorescence readings were measured after each shaking cycle. For αSyn-SAA, samples were tested in triplicate and assessed over 24 hours.

### Seed amplification assay data analysis

For both Tau and αSyn SAA reactions, individual wells were considered positive if they breached an empirically derived threshold of 1000 arbitrary units (au) in the assay run time. These thresholds were maintained for both brain tissue and CSF analysis. For the analysis of neat CSF, samples were deemed Tau-SAA positive if at least three out of five individual replicates surpassed the predefined 1000 au threshold. For αSyn-SAA analysis of neat CSF, samples were considered positive only if all three individual replicates exceeded the threshold fluorescence. Samples in which none or a single replicate breached the threshold were considered negative, and samples in which two of three replicates surpassed the threshold were deemed inconclusive and excluded from downstream analysis. The latter happened with only one sample in the αSyn-SAA test.

To derive semi-quantitative seeding scores for Tau-SAA brain and CSF seeding capacity as well as for αSyn-SAA seeding in brain tissues, we calculated the proportion of positive wells relative to total wells tested at that dilution. This process was repeated for all dilutions assessed and the final score was obtained by summing the proportions of positive wells for all dilutions.

### Statistical Analysis

All statistical analysis were performed in R (v4.4) utilizing freely available packages. Significance thresholds were set at α = 0.05. Group comparisons of seeding scores were assessed using Kruskal-Wallis tests, followed by Dunn’s post hoc and Benjamini-Hochberg correction for multiple comparisons. Pairwise comparisons restricted to two groups were assessed by Wilcoxon rank-sum test. Trends in seeding scores across ordered neuropathological stages (ADNC, NFT stage) were evaluated using the jonckheere.test() function from the *clinfun* package with p values computed by permutation (nperm = 10000). Associations between continuous variables were assessed using Pearson correlations, with r values, linear fits, and 95% confidence intervals shown as indicated. Categorical demographic comparisons (i.e sex distribution across groups) were performed using chi-squared test or Fisher’s exact test.

End-point titers or the seeding dose at which 50% of tested wells are positive were estimated using the Spearman-Kärber method for brain samples achieving a complete attack rate (100% of tested wells positive) at the lowest dilution tested (10^−4^). SD50 values were compared to tau brain seeding score values using Pearson correlation.

Diagnostic performance of CSF Tau-SAA was evaluated using receiver operating characteristic (ROC) curve analysis from the *pROC* R package. Area under the curve (AUC) and 95% confidence intervals are reported in Supplementary Fig 4B. Optimal positivity thresholds were determined by Youden’s index along with sensitivity, specificity, positive predictive value (PPV) and negative predictive value.

### Linear mixed model analysis

To evaluate the effect of αSyn on tau seeding activity in AD, we restricted analysis to AD group donors (*n = 34*) with tau seeding data available in brain and CSF. We used the αSyn-SAA result to dichotomize AD donors as αSyn-SAA+ or αSyn-SAA–. A linear mixed model was fit with tau seeding score as the outcome and matrix type (brain vs. CSF), CSF αSyn-SAA result, NFT stage, age at death (scaled), and sex included as fixed effects. The donor was included as a random intercept to account for within-donor correlation between matched brain and CSF measures. Models were fit using the lmer() function in the *lme4* package, with the fixed-effect *p* values computed using default Satterthwaite’s degrees of freedom approximation as implemented in *lmerTest*. Multicollinearity was assessed using variance inflation factors (VIF). All VIF values were below 1.5. Model assumptions were verified using DHARMa simulation-based residual diagnostics (uniformity, dispersion, outlier, and quantile deviation tests) and Shapiro-Wilk testing of random effect normality.

To assess whether the effect of αSyn positivity on tau seeding differed across matrices, a matrix type × CSF αSyn-SAA result interaction term was added to the model. As this interaction proved not significant, the additive model (without interaction) was retained for inference. As an additional sensitivity analysis, independent linear models were fit separately within each matrix (i.e. effect of αSyn-SAA positivity on tau seeding in the brain or CSF independently), regressing tau seeding score on CSF αSyn-SAA result, NFT stage, age at death, and sex.

Estimated marginal means for tau seeding score by CSF αSyn status, stratified by matrix type, were computed using the *emmeans* package. Partial residuals were derived by adjusting observed tau seeding scores for NFT stage, age at death, and sex. For visualization, tau seeding score partial residuals were anchored to the estimated marginal mean scale.

To assess the effect of tau on αSyn seeding capacity in synucleinopathy donors, we fit a linear model with αSyn-SAA brain seeding as the outcome and Tau-SAA brain seeding score and neuropathological diagnosis (MSA vs LBD) as covariates. Age at death and sex were evaluated as additional covariates but did not improve the model fit by Akaike information criterion. An analogous model substituting CSF Tau seeding score for brain seeding likewise showed no significant effect of tau seeding on αSyn seeding activity.

## Supplementary Files

This is a list of supplementary files associated with this preprint. Click to download.

- SupplementaryMaterials.pdf

## Figures and Tables

**Figure 1.**
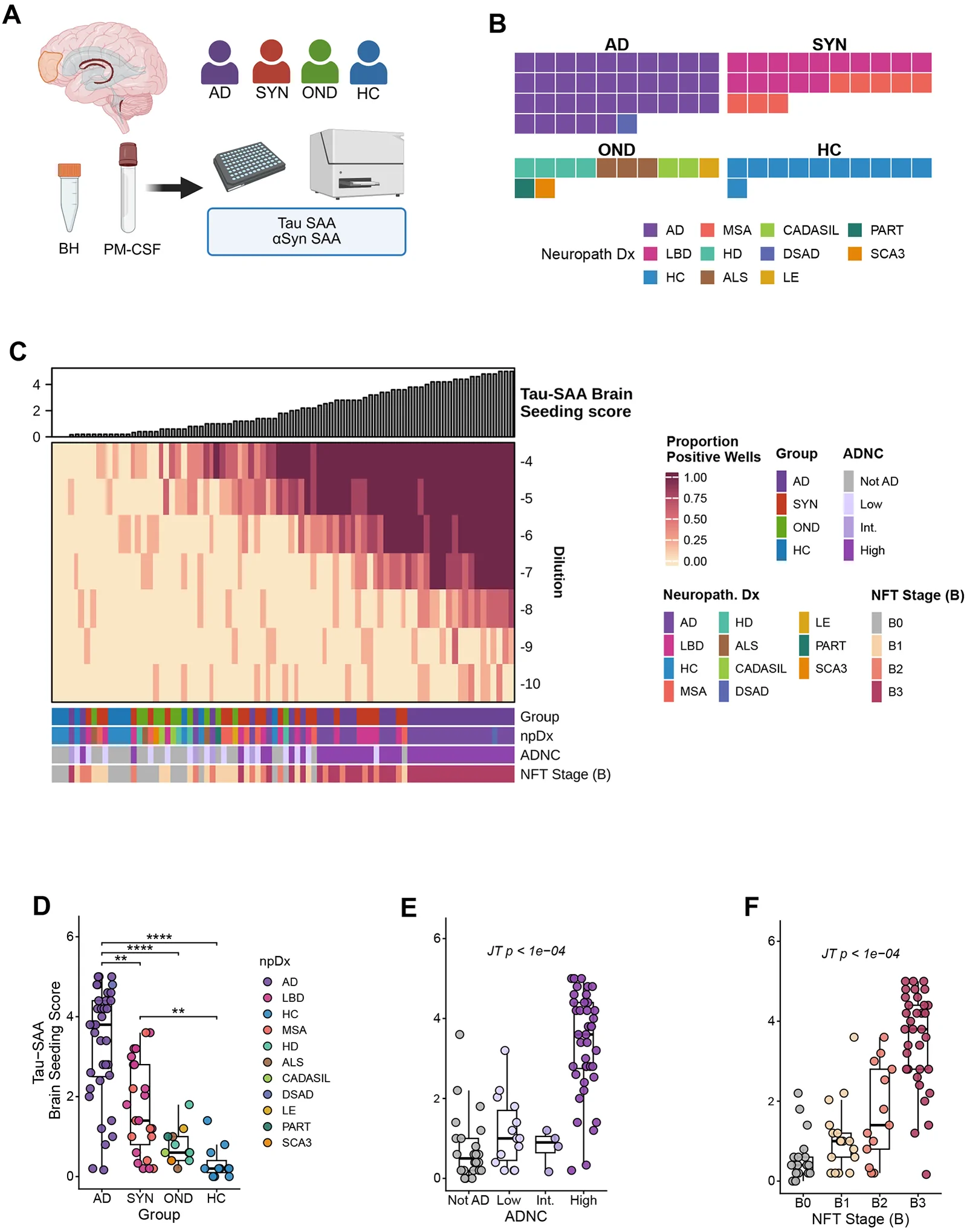
Brain Tau-SAA seeding activity reflects neuropathological diagnosis and disease severity. **(A)** Schematic of study design. **(B)** Waffle-plot of experimental groups (AD, *n* = 36, SYN, *n* = 23, OND, *n* = 12, HC, *n* = 11). Each tile represents an individual sample while colors represent the primary neuropathological diagnosis. **(C)** Heatmap of brain tissue Tau-SAA results. Columns indicate individual donors while rows indicate the ten-fold serial dilution (10^indicated^). Fill color denotes the proportion of replicates (*n* = 5–6) surpassing a predefined threshold (1000 au). Top bar plot annotation represents the tau seeding score for each sample, defined as summed proportion of positive wells across all tested dilutions (theoretical min = 0, max = 7). Bottom annotations indicate the experimental group, neuropathological diagnosis (npDx), Alzheimer’s disease neuropathological change (ADNC) and NFT stage (“B” score) based on postmortem brain evaluation following NIA-AA criteria. **(D)** Tau seeding score across groups. Points represent individual donors while fill color denotes the neuropathological diagnosis. Asterisk’s denote significance (**, *adj p* < 0.01; ****, *adj p* < 0.0001). Significance assessed by Kruskal-Wallis test (H(3) = 41.4, *p* = 5.36 × 10^−9) with Dunn’s post hoc test and Benjamini-Hochberg correction. Adjusted p-values for significant pairwise comparisons: AD vs. SYN, *p* = 0.002; AD vs. OND and AD vs. HC, *p* < 0.0001; SYN vs. HC, *p* = 0.007. **(E-F)** Tau seeding score stratified by **(E)** ADNC grade and **(F)** neurofibrillary tangle (NFT) stage. Statistical trends were assessed using Jonckheere-Terpstra test for ordered alternatives. Tau seeding score increased significantly with both ADNC grade (JT = 1861, *p* < 0.0001) and NFT stage (JT = 2049.5, *p* < 0.0001). **Abbreviations**: AD, Alzheimer’s disease; SYN, synucleinopathy; OND, other neurological disease; HC, healthy control; npDx, neuropathological diagnosis; ADNC, Alzheimer’s disease neuropathological change; LBD, Lewy body disease; MSA, multiple system atrophy; PD, Parkinson’s disease; HD, Huntington’s disease; ALS, amyotrophic lateral sclerosis; CADASIL, cerebral autosomal dominant arteriopathy with subcortical infarcts and leukoencephalopathy; LE, leukoencephalopathy; MS, multiple sclerosis; SCA3, spinocerebellar ataxia type 3; DSAD, Down syndrome Alzheimer’s disease; PART, primary age-related tauopathy; B, neurofibrillary tangle stage (Braak stage).

**Figure 2.**
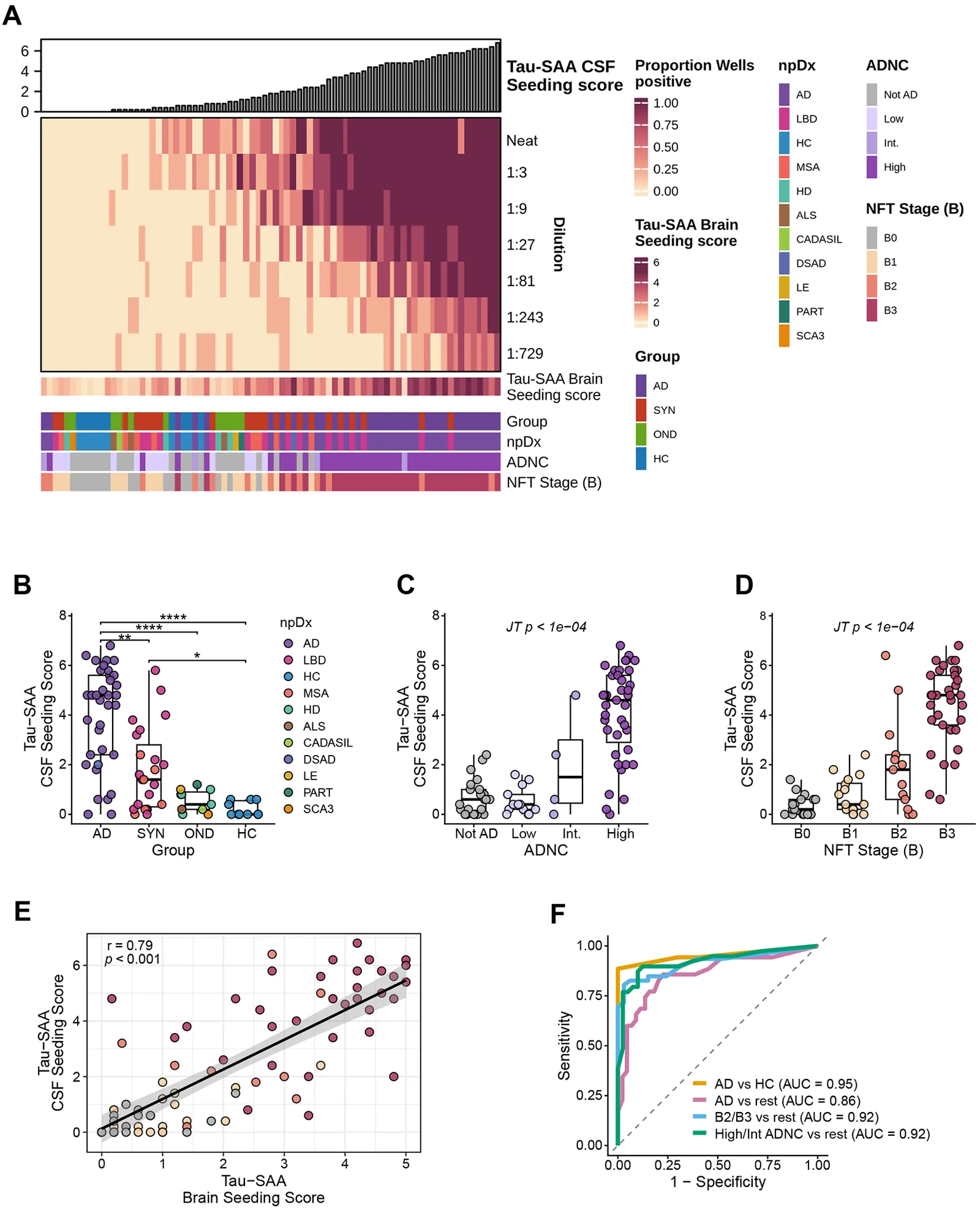
CSF Tau-SAA seeding activity mirrors neuropathology and correlates with brain Tau-SAA seeding **(A)** Heatmap of CSF Tau-SAA results. Columns indicate individual donors (*n* = 72) while rows indicate the dilution (denoted parts CSF: total parts). Fill color denotes the portion of replicates (*n* = 5) surpassing the 1000 au positivity threshold. Top bar plot represents the CSF tau seeding score (CSF Tau-SS) for each sample defined as the summed proportion of positive wells across all dilutions (theoretical min = 0, max = 7). Bottom annotations indicate experimental group, neuropathological diagnosis (npDx), ADNC grade and NFT stage. **(B)** CSF Tau-SS across groups (AD, *n* = 35; SYN, *n* = 23; OND, *n* = 11; HC, *n* = 10). Points represent individual donors while fill color denotes neuropathological diagnosis. Asterisks denote significance (*, *adj p* < 0.05; **, *adj p* < 0.01; ****, *adj p* < 0.0001). Significance assessed by Kruskal-Wallis test (H(3) = 36.9, *p* = 4.78 × 10^−8^) with Dunn’s post hoc test and Benjamini-Hochberg correction. Adjusted p-values for significant pairwise comparisons: AD vs. SYN, *p* = 0.002; AD vs. OND and AD vs. HC, *p* < 0.0001; SYN vs. HC, *p* = 0.02. **(C-D)** CSF Tau-SS stratified by **(C)** ADNC grade and **(D)** NFT stage (B score). Statistical trends assessed using the Jonckheere-Terpstra test for ordered alternatives. CSF Tau-SS increased significantly with both ADNC grade (JT = 1676.5, *p* < 0.0001) and NFT stage (JT = 1918.5, *p* < 0.0001). **(E)** Within-donor correlation between brain and CSF Tau-SS. Fill color denotes NFT stage. Line represents linear fit with 95% confidence interval (Pearson’s *r* = 0.79, *p* < 0.0001). **(F)** Receiver operating characteristic (ROC) curves for CSF Tau-SS discriminating AD from healthy controls (AUC = 0.95), from all other groups (AUC = 0.86), high/intermediate ADNC from low/none (AUC = 0.92), and advanced NFT stage (B2/B3) from early/absent (AUC = 0.92).

**Figure 3.**
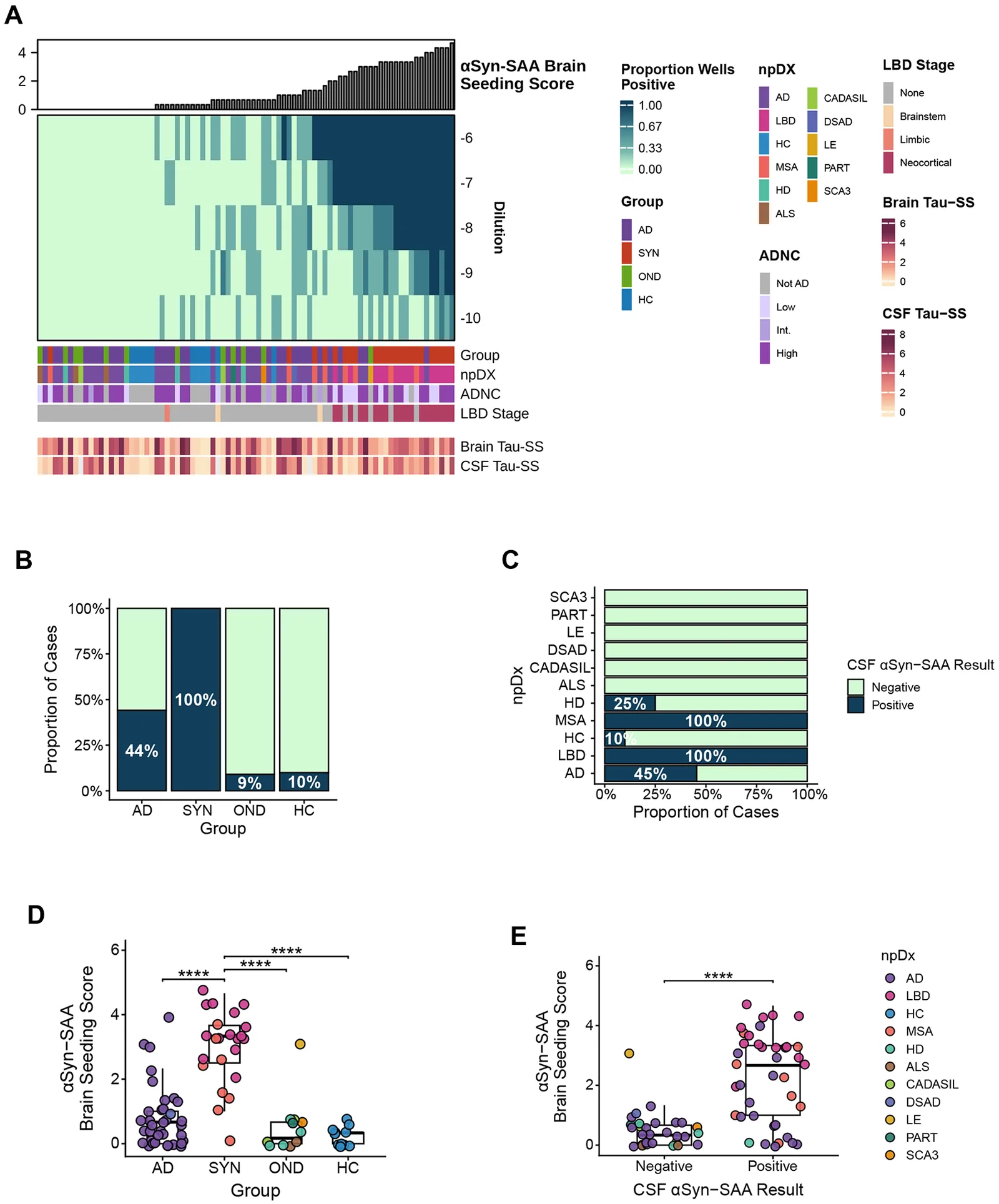
αSyn-SAA seeding activity in paired brain and CSF. **(A)** Heatmap of αSyn-SAA across brain homogenates seeded at 10^−6^ to 10^−10^ final dilutions. Columns indicate donors while rows indicate the final seed dilution. Columns are ordered by the αSyn-SAA seeding score (top bar plot annotation) derived by summing the proportion of positive wells across all tested dilutions. Bottom annotations denote the experimental group, neuropathological diagnosis (npDx), ADNC grade, LBD stage, and both brain and CSF tau seeding scores. **(B-C)** Proportion of αSyn-SAA positive donors across **(B)** groups and **(C)** neuropathological diagnosis. **(D)** Brain αSyn seeding scores (Brain α-Syn-SS) by experimental group. Comparisons were made by Kruskal-Wallis test with Dunn’s post hoc and Benjamini-Hochberg correction for multiple comparisons. **(E)** Brain αSyn-SS stratified by CSF αSyn-SAA result. Significance in E assessed by Wilcoxon rank-sum test. Significance annotations: **** *p* < 0.0001.

**Figure 4.**
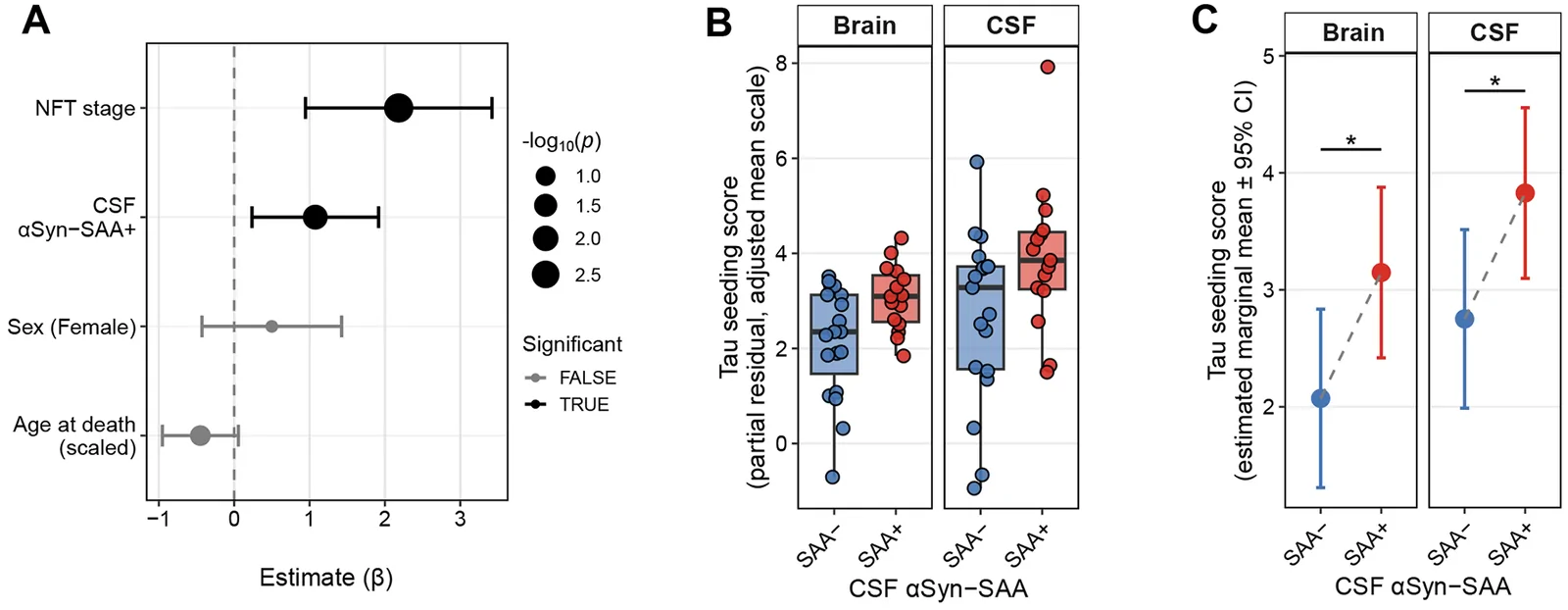
Linear mixed model analysis reveals effect of CSF αSyn-SAA positivity on tau seeding activity in Alzheimer’s Disease. **(A)** Forest plot depicting fixed effect estimates (β) with 95% confidence interval (CI; error bars) from linear mixed model examining predictors of tau seeding activity in AD group donors (*n* = 34). The model included the matrix type (brain vs CSF), the CSF αSyn-SAA result, NFT stage, age at death, and sex as fixed effects the donor as a random intercept. Point size reflects the −log_10_(*p*-value) with significant predictors (*p* < 0.05) shown in black and non-significant predictors in grey. The tau seeding matrix type was included as a structural covariate to account for scale differences and is not shown. **(B)** Tau seeding partial residuals for brain and CSF stratified by CSF αSyn-SAA result. Values represent observed tau seeding scores adjusted for NFT stage, age at death and sex. Values are anchored to the estimated marginal means scale. Points represent individual donors while boxes denote the group median (midline) and interquartile range. **(C)** Estimated marginal means (± 95% CI) for brain and CSF stratified by CSF αSyn-SAA result. αSyn-SAA positive (SAA+) donors showed significantly higher tau seeding activity relative to negative donors (SAA-) in both brain and CSF (β = 1.08, *p* = 0.014). Model assumptions were verified using DHARMa simulation-based diagnostics (uniformity *p* = 0.681, dispersion *p* = 0.632, outliers *p* = 0.420, quantile deviation *p* = 0.109), and Shapiro Wilk test of random effect normality (W = 0.981, *p* = 0.791).

**Figure 5.**
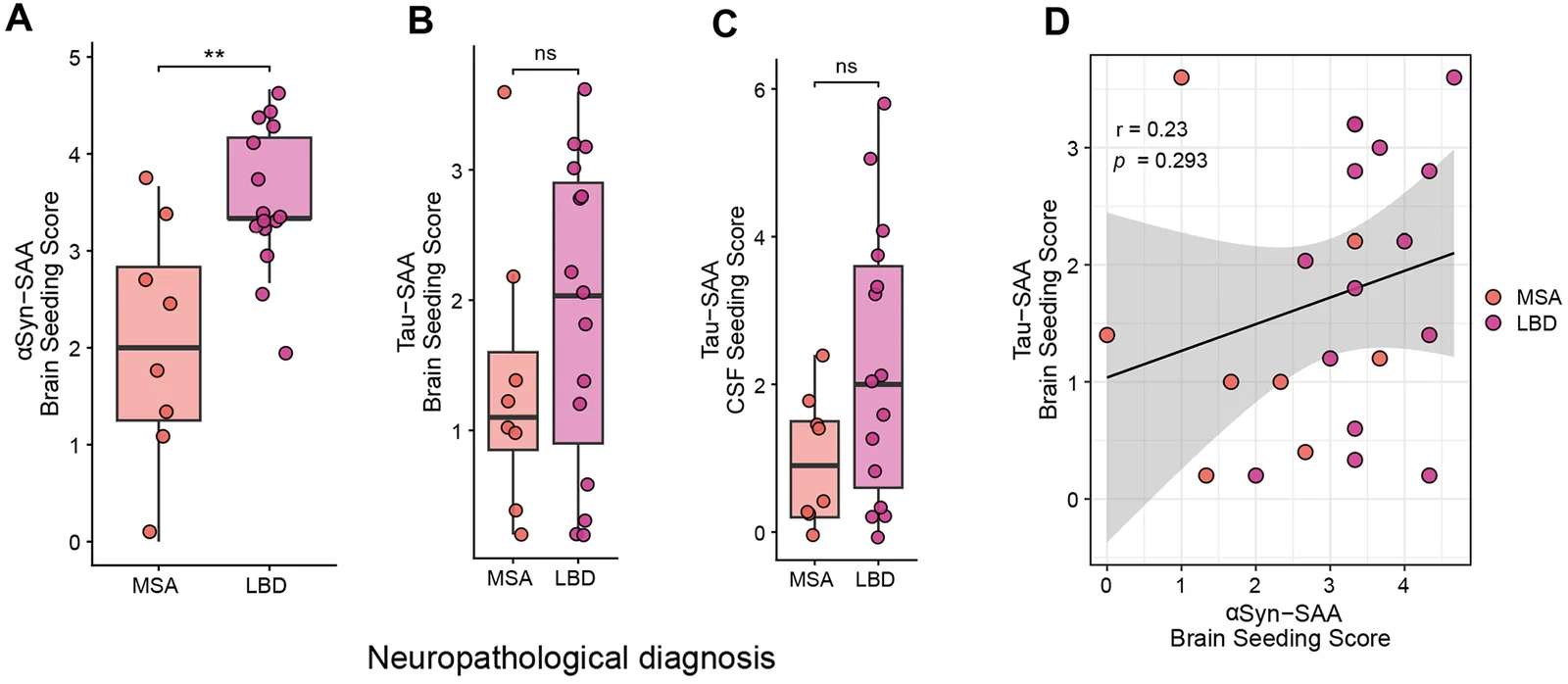
αSyn seeding capacity differs between MSA and LBD but is not predicted by concomitant tau **(A)** Brain αSyn-SAA seeding scores in Syn group donors stratified by neuropathological diagnosis (MSA, *n* = 8, LBD, *n* = 15) demonstrating significantly higher seeding in LBD compared to MSA donors (Wilcoxon *p* = 0.005). **(B)** Brain and **(C)** CSF tau seeding scores across MSA and LBD donors. No significant differences in tau seeding were observed across brain (Wilcoxon *p* = 0.365) or CSF matrices (Wilcoxon *p* = 0.128). **(D)** Correlation between brain αSyn-SAA and Tau-SAA seeding scores. Points denote individual donors colored by their neuropathological diagnosis. Shaded ribbon indicates 95% confidence interval. No significant correlation was observed between tau and αSyn seeding activity (Pearson’s *r*, *p* = 0.293)

## Data Availability

Data will be deposited in a freely accessible repository at the time of publication. All code utilized for analysis and figure generation is available at the following GitHub repository: https://github.com/dgorski147/tau-asyn-saa-panel.
